# Delayed gastric emptying is associated with increased risk of mortality in patients undergoing pancreaticoduodenectomy for pancreatic adenocarcinoma

**DOI:** 10.1007/s13304-022-01404-4

**Published:** 2022-10-30

**Authors:** Oscar Hernandez Dominguez, Areg Grigorian, Ronald F. Wolf, David K. Imagawa, Jeffry T. Nahmias, Zeljka Jutric

**Affiliations:** Division of Hepatobiliary and Pancreas Surgery and Islet Cell Transplantation, Department of Surgery, Irvine Medical Center, University of California, 333 The City Blvd West, Suite 1600, Orange, CA 92868-3298 USA

**Keywords:** Pancreaticoduodenectomy, Pancreatic fistula, Delayed gastric emptying, Pancreatic cancer

## Abstract

Delayed gastric emptying (DGE) is common in patients undergoing pancreaticoduodenectomy (PD). The effect of DGE on mortality is less clear. We sought to identify predictors of mortality in patients undergoing PD for pancreatic adenocarcinoma hypothesizing DGE to independently increase risk of 30-day mortality. The ACS-NSQIP targeted pancreatectomy database (2014–2017) was queried for patients with pancreatic adenocarcinoma undergoing PD. A multivariable logistic regression analysis was performed. Separate sensitivity analyses were performed adjusting for postoperative pancreatic fistula (POPF) grades A–C. Out of 8011 patients undergoing PD, 1246 had DGE (15.6%). About 8.5% of patients with DGE had no oral intake by postoperative day-14. The DGE group had a longer median operative duration (373 vs. 362 min, *p* = 0.019), and a longer hospital length of stay (16.5 vs. 8 days, *p* < 0.001). After adjusting for age, gender, comorbidities, preoperative chemotherapy, preoperative radiation, open versus laparoscopic approach, vascular resection, deep surgical space infection (DSSI), postoperative percutaneous drain placement, and development of a POPF, DGE was associated with an increased risk for 30-day mortality (OR 3.25, 2.16–4.88, *p* < 0.001). On sub-analysis, grades A and B POPF were not associated with risk of mortality while grade C POPF was associated with increased risk of mortality (OR 5.64, 2.24–14.17, *p* < 0.001). The rate of DGE in patients undergoing PD in this large database was over 15%. DGE is associated with greater than three times the increased associated risk of mortality, even when controlling for POPF, DSSI, and other known predictors of mortality.

## Introduction

Pancreatic ductal adenocarcinoma (PAC) is the third-leading cause of cancer death in the United States with only 11.5% surviving 5 years after diagnosis [1]. Surgical resection remains the foundation of treatment for non-metastatic PAC [2]. The perioperative mortality of patients undergoing pancreaticoduodenectomy (PD) has decreased to 1–4% [3, 4]. Despite significant improvement in mortality, perioperative morbidity remains substantial. Studies report morbidity rates from 30 to 60%, with common postoperative complications consisting of delayed gastric emptying (DGE) and postoperative pancreatic fistula (POPF) [3, 5].

DGE has been observed to be a complication in a high number of patients (up to 40%) undergoing PD, but whether DGE itself is associated with risk of mortality in PD patients remains unclear [6–8]. In contrast, POPF occurs in close to 20% of patients undergoing PD and has been shown to be associated with an increased risk of mortality [7, 9, 10]. Many studies have recognized the strong relationship between POPF and the occurrence of DGE [11–15], and have proposed that POPF grade C is an independent risk factor for DGE. [16]

This study aimed to assess the risk of mortality in patients with DGE, when controlling for POPF and other significant predictors of mortality, with a hypothesis that DGE is independently associated with an increased risk of 30-day mortality.

## Methods

A retrospective analysis of the American College of Surgeons-National Surgery Quality Improvement Program (ACS-NSQIP) targeted pancreatectomy database (2014–2017) was performed. All patients with PAC undergoing PD were identified. Patients with documented DGE were compared to patients without DGE. DGE was defined by NSQIP parameters as no oral intake by postoperative day fourteen or tube to external drainage, or if a nasogastric tube was present or reinserted [17].

The primary outcome was a 30-day mortality. Other outcomes measured were NSQIP-defined postoperative complications: bleeding requiring transfusions > 4 units, organ surgical site infection, deep surgical site infection, surgical site infection, sepsis, septic shock, unplanned intubation, ventilation ≥ 48 h, pneumonia, deep vein thrombosis, urinary tract infection, dehiscence, *Clostridium difficile* infection, acute renal failure, cardiac arrest with cardiopulmonary resuscitation, myocardial infarction, renal insufficiency, pulmonary embolism, and cerebrovascular accident. Additional outcomes measured included total hospital length of stay (LOS) and intensive care unit (ICU) LOS. The demographic variables collected included age and gender.

Frequency statistics were performed for all available variables. A Student’s *t* test or Mann–Whitney-*U* test was used to compare continuous variables, and chi-square was used to compare categorical variables for bivariate analysis. Continuous data were reported as medians with interquartile range or as means with standard deviation. Categorical data were reported as percentages.

The magnitude of the association between predictor variables and mortality was first measured using a univariable logistic regression model. Covariates were chosen by author consensus and review of the literature and included sex, age greater than 65, bleeding disorder, congestive heart failure, chronic obstructive pulmonary disease (COPD), diabetes mellitus, hypertension, smoker, steroid use, weight loss, preoperative chemotherapy, preoperative radiation, vascular resection, percutaneous drain, deep surgical site infection, open operative approach, DGE, and development of POPF [18–26]. Covariates with statistical significance (*p* < 0.20) were included in a hierarchical multivariable logistic regression model and the adjusted risk for mortality was reported with an odds ratio (OR) and 95% confidence intervals (CI). Several studies have reported POPF to be associated with increased risk of DGE and associated with the development of clinical DGE [11–16]. Therefore, a separate sensitivity analysis adjusting for POPF grades A-C was performed because POPF could be a significant confounder of our analysis. NSQIP defines POPF as either a clinical diagnosis and NPO-TPN, drain continued longer than 7 days, percutaneous drainage, spontaneous wound drainage, or reoperation; or as persistent drain output of amylase rich fluid in combination with NPO-TPN, a drain continued longer than 7 days, percutaneous drainage, spontaneous wound drainage, or reoperation. NSQIP POPF grades were defined according to the International Study Group on Pancreatic Fistula (ISGPF) guidelines [27]. All *p* values were two-sided, with a statistical significance level of < 0.05. All analyses were performed with IBM SPSS Statistics for Windows (Version 24, IBM Corp., Armonk, NY).

## Results

### Demographics and operative characteristics

From 8011 patients undergoing PD for PAC, 1246 developed DGE (15.6%) and 6765 did not (84.4%). Compared to patients without DGE, patients with DGE were more often male (62.0% vs. 52.1%, *p* < 0.001) and older (median, 69 vs. 67-years-old, *p* < 0.001). Patients with DGE had higher rates of hypertension (56.7% vs. 52.7%, *p* = 0.008) and COPD (6.4% vs. 3.9%, *p* < 0.001). Patients with DGE were less likely to have received chemotherapy (23.5% vs. 29.2, *p* < 0.001) or radiation therapy within 90 days preoperatively (10.0% vs. 12.4%, *p* = 0.006), compared to patients without DGE Table 1.

**Table 1:** Demographics of patients undergoing pancreaticoduodenectomy stratified by delayed gastric emptying

	DGE	No DGE	
Characteristic	(*n* = 1246)	(*n *= 6765)	*P* value
Age, year, median (IQR)	69.0 (61, 75)	67.0 (60, 73)	< 0.001
Male, *n* (%)	773 (62.0%)	3522 (52.1%)	< 0.001
Comorbidities, *n* (%)			
Hypertension	707 (56.7%)	3563 (52.7%)	0.008
Diabetes mellitus	350 (28.1%)	1916 (28.3%)	0.867
Weight loss	233 (18.7%)	1325 (19.6%)	0.468
Smoker	193 (15.5%)	1197 (17.7%)	0.059
COPD	80 (6.4%)	264 (3.9%)	< 0.001
Ascites	8 (0.6%)	17 (0.3%)	0.023
Bleeding disorder	41 (3.3%)	198 (2.9%)	0.488
Steroid	31 (2.5%)	148 (2.2%)	0.510
Congestive heart failure	2 (0.2%)	30 (0.4%)	0.146
End-stage-renal disease	2 (0.2%)	8 (0.1%)	0.698
Functional health status prior to surgery, *n* (%)			
Independent	1231 (98.8%)	6713 (99.2%)	0.308
Partially dependent	1 (0.9%)	1 (0.6%)	0.308
Totally dependent	1 (0.1%)	1 (0.0%)	0.308
Preoperative obstructive jaundice, *n* (%)	703 (56.4%)	3858 (57.0%)	0.919
Chemotherapy within 90 days, *n* (%)	293 (23.5%)	1976 (29.2%)	< 0.001
Radiation therapy within 90 days, *n* (%)	125 (10.0%)	839 (12.4%)	0.006

*DGE* Delayed Gastric Emptying, *IQR* interquartile range, *COPD* chronic obstructive pulmonary disease

Patients that developed DGE after PD had higher rates of POPF grade A (16.9% vs. 6.6%, *p* < 0.001), POPF grade B (14.2% vs. 3.5%, *p* < 0.001), and POPF grade C (4.0% vs. 0.5%, *p* < 0.001) compared to patients without DGE. Additionally, patients with DGE had higher rates of postoperative percutaneous drain placement compared to patients without DGE (18.9% vs. 8.2%, *p* < 0.001). There was no difference in the operative approach and vascular resection when comparing patients that developed DGE after PD compared to those without DGE Table 2. The type of anastomosis and its effect on developing DGE could not be studied in more detail given that this was a static variable in the database and the type of anastomosis each patient underwent could not be separated.

**Table 2:** Operative characteristics of patients undergoing pancreaticoduodenectomy stratified by delayed gastric emptying

	DGE	No DGE	
Characteristic	(*n* = 1246)	(*n* = 6765)	*P* value
Operative approach, *n* (%)			
Open (planned)	1144 (91.8%)	6247 (92.3%)	0.140
Hybrid	7 (0.4%)	16 (0.2%)	0.140
Laparoscopic	44 (0.9%)	250 (1.7%)	0.140
Robotic	51 (3.3%)	249 (3.0%)	0.140
Pancreatic duct size, *n* (%)			
< 3 mm	254 (20.4%)	1260 (18.6%)	0.052
3–6 mm	528 (42.4%)	3050 (45.1%)	0.052
> 6 mm	168 (13.5%)	1010 (14.9%)	0.052
Pancreatic gland texture, *n* (%)			
Hard	459 (36.8%)	2842 (42.0%)	< 0.001
Intermediate	114 (9.1%)	665 (9.8%)	< 0.001
Soft	361 (29.0%)	1572 (23.2%)	< 0.001
G-J or D-J anastomoses, *n* (%)			
Antecolic fashion	305 (24.5%)	1771 (26.2%)	< 0.001
Retrocolic fashion	136 (10.9%)	743 (11.0%)	< 0.001
Not performed	22 (1.8%)	63 (0.9%)	< 0.001
Vascular resection, *n* (%)			
Artery	27 (2.2%)	126 (1.9%)	0.248
Vein	224 (18.0%)	1144 (16.9%)	0.248
Vein and artery	43 (3.5%)	263 (3.9%)	0.248
Not performed	934 (75.0%)	5172 (76.5%)	0.248
Drain type, *n* (%)			
Biliary anastomosis	13 (1.0%)	74 (1.1%)	0.007
Pancreatic and biliary anastomosis	359 (28.8%)	1774 (26.2%)	0.007
Pancreatic anastomosis	92 (7.4%)	429 (6.3%)	0.007
Pancreatic parenchyma	12 (1.0%)	40 (0.6%)	0.007
Type(s) cannot be determined	59 (4.7%)	234 (3.5%)	0.007
Drains, *n* (%)	1120 (89.9%)	5862 (86.7%)	0.007
Percutaneous drain, *n* (%)	236 (18.9%)	555 (8.2%)	< 0.001
Pancreatic fistula A, *n* (%)	177 (16.9%)	425 (6.6%)	< 0.001
Pancreatic fistula B, n (%)	144 (14.2%)	218 (3.5%)	< 0.001
Pancreatic fistula C, n (%)	36 (4.0%)	29 (0.5%)	< 0.001
Pancreatic fistula, n (%)			
CD, Drain continued > 7 days	33 (2.6%)	98 (1.4%)	< 0.001
CD, NPO-TPN	15 (1.2%)	11 (0.2%)	< 0.001
CD, Percutaneous drainage performed	57 (4.6%)	102 (1.5%)	< 0.001
CD, Reoperation performed	22 (1.8%)	23 (0.3%)	< 0.001
CD, Spontaneous wound drainage	12 (1.0%)	20 (0.3%)	< 0.001
PD, Drain continued > 7 days	144 (11.6%)	327 (4.8%)	< 0.001
PD, NPO-TPN	19 (1.5%)	11 (0.2%)	< 0.001
PD, percutaneous drainage performed	41 (3.3%)	74 (1.1%)	< 0.001
PD, reoperation performed	14 (1.1%)	6 (0.1%)	< 0.001

*DGE* Delayed Gastric Emptying, *N/A*  Not available, *G-J* Gastrojejunal, *D-J* Duodenojejunal, *CD* Clinical Diagnosis, *PD* Persistent Drainage, *NPO* Nothing by mouth, *TPN* total parenteral nutrition

### Predictors of 30-day mortality for PAC patients undergoing PD

On multivariable analysis, DGE was independently associated with increased risk for 30-day mortality (OR 3.25, 2.16–4.88, *p* < 0.001). Additional associated risk factors for 30-day mortality included: development of POPF (OR 2.31, 1.44–3.70, *p* < 0.001), vascular resection (OR 1.83, 1.21–2.78, *p* = 0.005), hypertension (OR 1.62, 1.05–2.49, *p* = 0.039), and age ≥ 65-years-old (OR 1.61, 1.02–2.53, *p* < 0.001) Table 3. Preoperative radiation/chemotherapy, sex, diabetes, COPD were not associated with 30-day mortality (all *p* > 0.05) Table 3. On sub-analysis, grades A and B POPFs were not associated with risk of mortality while grade C POPFs were associated with an increased risk of mortality (OR 5.64, 2.24–14.17, *p* < 0.001) Table 4.

**Table 3:** Adjusted^*^ risk of mortality for patients undergoing pancreaticoduodenectomy

Risk factor	OR	CI	*P* value
Delayed gastric emptying	3.25	2.16–4.88	< 0.001
Development of pancreatic fistula	2.31	1.44–3.70	< 0.001
Steroid	1.86	0.73–4.77	0.196
Vascular resection	1.83	1.21–2.78	0.005
COPD	1.64	0.82–3.28	0.163
Hypertension	1.62	1.05–2.49	0.028
Age ≥ 65	1.61	1.02–2.53	0.039
Pre-op radiation	1.49	0.74–3.01	0.268
Organ SSIs	1.49	0.84–2.62	0.171
Gender	1.23	0.82–1.83	0.314
Bleeding disorder	1.28	0.51–3.21	0.605
Smoker	1.13	0.67–1.92	0.643
Diabetes	1.12	0.73–1.70	0.613
Pre-op weight loss	0.97	0.59–1.60	0.899
Post-op percutaneous drain placement	0.90	0.48–1.67	0.727
Pre-op chemotherapy	0.79	0.45–1.36	0.388
Open operative approach	0.68	0.37–1.26	0.217
Congestive heart failure	0.00	0.00–0.00	0.998

*COPD* chronic obstructive pulmonary disease, *Pre-op* preoperative, *Post-op* postoperative, *SSIs* surgical site infection

**Table 4:** Adjusted^*^ risk of mortality for pancreaticoduodenectomy adjusting for pancreatic fistula grades

Risk factor	OR	CI	*P* value
Pancreatic fistula A	1.619	0.87–3.01	0.127
Pancreatic fistula B	1.538	0.67–3.54	0.311
Pancreatic fistula C	5.635	2.24–14.17	< 0.001

^*^controlled for: delayed gastric emptying, age > 65, gender, bleeding disorder, congestive heart failure, chronic obstructive pulmonary disease, diabetes mellitus, hypertension, smoker, steroid use, weight loss, preoperative chemotherapy, preoperative radiation, vascular resection, percutaneous drain, deep organ surgical site infection, open operative approach and pancreatic fistula grade (A, B,C)

### Other clinical outcomes in PAC patients undergoing PD

Patients that developed DGE had longer operative duration (373 vs. 362 min, *p* = 0.019), longer LOS (16.5 vs. 8 days, *p* < 0.001), and greater rate of readmission within 30-days (24.2% vs. 13.3%, *p* < 0.001) when compared to patients without DGE. Patients with DGE also had higher rates of packed red blood cell transfusions (28.1% vs. 20.0%, *p* < 0.001), organ surgical site infections (24.0% vs. 9.3%, *p* < 0.001), sepsis (13.8% vs. 7.3%, *p* < 0.001), unplanned intubation (10.4% vs. 2.1%, *p* < 0.001) and mortality (4.0% vs. 0.9%, *p* < 0.001), as compared to patients without DGE Table 5.

**Table 5:** Clinical outcomes in patients undergoing pancreaticoduodenectomy stratified by delayed gastric emptying

Outcome	DGE (*n* = 1246)	No DGE (n = 6765)	*P* value
Total operation time, minutes, median (IQR)	373 (297.0, 464.5)	362 (282.0, 447.0)	0.019
LOS, days, median (IQR)	16.5 (11, 23)	8.0 (6, 10)	< 0.001
30-day Readmission, *n* (%)	301 (24.2%)	897 (13.3%)	< 0.001
30-day Complications, *n* (%)			
Transfusion	350 (28.1%)	1355 (20.0%)	< 0.001
Organ surgical site infection	299 (24.0%)	630 (9.3%)	< 0.001
Sepsis	172 (13.8%)	495 (7.3%)	< 0.001
Surgical site infection	148 (11.9%)	523 (7.7%)	< 0.001
Unplanned intubation	130 (10.4%)	141 (2.1%)	< 0.001
Ventilator past 48 h	126 (10.1%)	86 (1.3%)	< 0.001
Shock	123 (9.9%)	96 (1.4%)	< 0.001
Pneumonia	111 (8.9%)	156 (2.3%)	< 0.001
DVT	88 (7.1%)	151 (2.2%)	< 0.001
UTI	57 (4.6%)	176 (2.6%)	< 0.001
Dehiscence	41 (3.3%)	44 (0.7%)	< 0.001
C. difficile	26 (3.2%)	84 (1.8%)	0.009
Renal failure	31 (2.5%)	34 (0.5%)	< 0.001
Cardiac arrest with CPR	29 (2.3%)	58 (0.9%)	< 0.001
Myocardial infarction	29 (2.3%)	56 (0.8%)	< 0.001
Deep surgical site infection	28 (2.2%)	80 (1.2%)	0.003
Renal insufficiency	22 (1.8%)	18 (0.3%)	< 0.001
Pulmonary embolism	23 (1.8%)	58 (0.9%)	0.001
Cerebrovascular accident	8 (0.6%)	16 (0.2%)	0.016
Mortality, *n* (%)	50 (4.0%)	62 (0.9%)	< 0.001

*LOS* length of total hospital stay, *ICU* intensive care unit, *CPR* cardiopulmonary resuscitation, C. difficile = Clostridium difficile, *DVT* deep vein thrombosis, *UTI* urinary tract infection, *OR* operating room

### Independent predictors of developing DGE

PF is the highest risk factor for developing DGE (OR 2.71, 2.30–3.19, *p* < 0.001). Deep organ space infection was the second highest risk factor for developing DGE (OR 1.97, 1.66–2.35, *p* < 0.001), likely a surrogate measure of PF. Other risk factors included chronic obstructive pulmonary disease (OR 1.63, 1.25–2.14, *p* < 0.001), age > 65 years old (OR 1.32, 1.15–1.50, *p* < 0.001) and gender (OR 1.42, 1.25–1.62, *p* < 0.001).

## Discussion

High rates of postoperative morbidity remain a concern for patients undergoing PD. In this retrospective analysis spanning three years, we found that DGE was independently associated with an increased risk of 30-day mortality in patients that had undergone PD for PAC. Other predictors of 30-day mortality included vascular resection, age over 65 years and hypertension. Sex, comorbidities, preoperative chemotherapy, preoperative radiation, and operative approach were not associated with 30-day mortality risk. In a separate sensitivity analysis, we found that grade C POPF was associated with a greater than five-fold increase in associated risk of mortality.

DGE is a leading postoperative complication following PD, with a high reported incidence of up to 25% [3, 6–8]. Many studies have demonstrated an association of DGE with increased hospital LOS, readmission, delayed adjuvant therapy, and other complications [7, 11, 13, 15]. None of these studies found an independent association of DGE with mortality when controlling for other significant predictors of mortality following PD. Of the retrospective studies identifying postoperative PD mortality risk factors [6, 19–22], one study found DGE to be an independent risk factor for long-term pancreatic cancer survival [28]. The results of our current study demonstrate that DGE was independently associated with an increased risk of 30-day mortality following PD for PAC. This is not consistent with the ISGPS guidelines, which state that DGE is not a life-threatening complication [29]. A systematic review of clinical risk factors of DGE by Qu et al., also concluded that DGE was not life-threatening but rather a warning of other severe postoperative complications [11]. The results of the current study suggest that DGE may be addressed as an independent risk factor of mortality, rather than an association with other complications.

Multiple studies have identified risk factors of mortality following PD. Current literature shows that POPF [7, 9, 21] and hypertension [19, 22] are associated with an increased risk of mortality, while surgical approach has no significant difference in mortality [25, 30]. Factors such as age [19, 22] vascular resection [21, 31] comorbidities (COPD, diabetes, smoking) [19, 22] neoadjuvant chemotherapy/radiation [18] and gender [19] have received mixed association with risk of mortality following PD and our study follows this trend. In the current study, we found that that POPF grade C, vascular resection, hypertension, and age greater than 65 years were associated with an increased risk of 30-day mortality. These results are consistent with several studies which demonstrated an association of POPF with mortality [19, 21, 22, 31]. In regards to vascular resection, the results of this study add to the published literature demonstrating an increased risk of 30-day mortality [26, 32, 33]. When evaluating outcomes, the risk associated with hypertension, age greater than 65, and vascular resection should be incorporated into clinician and patient decision making regarding PD for PAC.

POPF remains a common and problematic complication following PD. Several retrospective studies have found Grade C POPF with a mortality rate as high as 30% after PD [7, 9]. However these studies did not control for DGE. Our study adds to existing literature with the finding that grade C POPF was associated with a greater than five-fold increase in risk of 30-day mortality for patients undergoing PD for PDAC, even when controlling for DGE. This reinforces that further work addressing reduction of high-grade pancreatic fistula is needed.

Our study has several limitations including those associated with a retrospective database analysis such as reporting bias, missing data, and misclassification although attempts are made by the ACS to limit these by stringent training requirements of the institutional coders that contribute data to NSQIP. Also, ACS-NSQIP is confined to 30-day outcomes; thus, long-term outcomes are not available. Finally, a significant limitation is that NSQIP does not use the ISGPS definition of DGE, making the definition non uniform with other studies. As such, we were unable to determine if DGE occurred independent of intra-abdominal complications versus concomitant; however, we controlled for these possible confounders during our analyses. Another limitation specific to our study is the inability to separately study the type of anastomosis created for each patient (gastrojejunostomy versus duodenojejunostomy, antecolic versus retrocolic) and its relationship with the development of DGE. Despite these limitations, our study is strengthened by the extensive library of clinical outcomes and variables specific to pancreatic surgery available in this targeted version of the NSQIP database.

## Conclusion

In summary, this study utilizing NSQIP found that for patients undergoing PD for PAC, DGE was independently associated with greater than three times the risk for 30-day mortality. In addition, POPF grade C, vascular resection, hypertension, and age greater than 65 years were associated with an increased risk of mortality. Specifically grade C POPF was associated with a greater than five times increased risk for 30-day mortality when controlling for DGE. We propose that DGE should be recognized as an independent risk factor for mortality. Future studies on reduction of occurrence of DGE are warranted.

## Declarations

### Research involved animals and humans

This research involved humans. However, since this retrospective study was performed using a national database with deidentified patients, risk to participants is minimal. There is no consent required.
